# Target margin design through analyzing a large cohort of clinical log data in the cyberknife system

**DOI:** 10.1002/acm2.13476

**Published:** 2022-01-19

**Authors:** Payam Samadi Miandoab, Shahyar Saramad, Saeed Setayeshi

**Affiliations:** ^1^ Department of Medical Radiation Engineering Amirkabir University of Technology Tehran Iran

**Keywords:** Cyberknife system, errors, PTV margins, uncertainties

## Abstract

**Purpose:**

Calculating the adequate target margin for real‐time tumor tracking using the Cyberknife system is a challenging issue since different sources of error exist. In this study, the clinical log data of the Cyberknife system were analyzed to adequately quantify the planned target volume (PTV) margins of tumors located in the lung and abdomen regions.

**Methods:**

In this study, 45 patients treated with the Cyberknife module were examined. In this context, adequate PTV margins were estimated based on the Van Herk formulation and the uncertainty estimation method by considering the impact of errors and uncertainties. To investigate the impact of errors and uncertainties on the estimated PTV margins, a statistical analysis was also performed.

**Results:**

Our study demonstrates five different sources of errors, including segmentation, deformation, correlation, prediction, and targeting errors, which were identified as the main sources of error in the Cyberknife system. Furthermore, the clinical evaluation of the current study reveals that the two different formalisms provided almost identical PTV margin estimates. Additionally, 4–5 mm and 5 mm margins on average could provide adequate PTV margins at lung and abdomen tumors in all three directions, respectively. Overall, it was found that concerning the PTV margins, the impact of correlation and prediction errors is very high, while the impact of robotics error is low.

**Conclusions:**

The current study can address two limitations in previous researches, namely insufficient sample sites and a smaller number of patients. A comparison of the present results concerning the lung and abdomen areas with other studies reveals that the proposed strategy could provide a better reference in selection the PTV margins. To our knowledge, this study is one of the first attempts to estimate the PTV margins in the lung and abdomen regions for a large cohort of patients treated using the Cyberknife system.

## 1 INTRODUCTION

Radiation therapy is a non‐invasive treatment option for patients with lung cancer that cannot undergo surgery.^1^ Successful conformal radiation therapy involves a uniform three‐dimensional (3D) dose distribution to the tumor volume, with only a minimum dose received by the surrounding tissue.^1^ However, tumor motion, which is affected by the respiratory motion, is highly challenging when hypo‐fractionated high‐dose radiotherapy, such as stereotactic body radiation therapy (SBRT), is proposed. To compensate for tumor motion in the absence of real‐time target localization and tracking, an additional internal margin is proposed to include the target movement. However, this approach, in particular, leads to a greater internal target volume (ITV) and increases the dose that is received by the surrounding tissue.^2^, ^3^, ^4^ Therefore, the internal margin size must be reduced in order to improve the accuracy of the delivered doses in the SBRT.^2^, ^5^ For this purpose, suitable techniques are needed to manage the respiratory motion, such as deep inspiration breath‐hold, respiratory gating, and real‐time tumor tracking methods. Among the available modern radiotherapy techniques, one of the best options, which can provide high‐dose accuracy during SBRT, is the Cyberknife system (Accuray, Inc., Sunnyvale, CA, USA).^3^ This system includes a 6 MeV mobile linear accelerator mounted on a highly maneuverable robotic arm, which is capable to move with 6 degrees of freedom (6‐DOF).^6^ This system by using the X‐ray image guidance, which is called synchrony,^7^, ^8^, ^9^ tracks the dynamic tumors which are affected by the patient's respiration. The target localization and tracking, based on the synchrony module, can be performed either through fiducial‐based target tracking (FTT) or the Xsight lung tracking (XLT) system. While the FTT tracking requires fiducial markers placed inside or near the tumor volume, the XLT tracking does not need any fiducial markers, allowing direct tracking of the tumor. This allows the patient to breathe normally during the treatment, while the robot arm moves continuously around the selected nodes to deliver an isocentric or non‐isocentric radiation beam.^7^, ^8^, ^9^, ^10^

In order to classify the sources of error for estimating the adequate target margins during the treatment with the Cyberknife system, studies on both types of respiratory tracking systems have been performed.^11^, ^12^, ^13^, ^14^, ^15^, ^16^, ^17^, ^18^, ^19^, ^20^ The review of some clinical studies showed that to estimate the planned target volume (PTV) margins, it is necessary to consider the correlation and prediction errors.^11^, ^18^, ^21^ A recent report from Yang et al. showed that segmentation, deformation, correlation, prediction, and targeting errors are the main sources of error in the respiratory tracking system.^9^ Based on the results of this study, the PTV margins in the lung area were estimated by analyzing the clinical log data of the XLT system according to Van Herk formula and the uncertainty estimation method. However, the value of the segmentation and deformation errors that were taken into account was based on previous studies.^9^ Essentially, the review of earlier studies also reveals the challenges faced by researchers, including insufficient sample sites, lower numbers of cases, and lack of true value for the segmentation or deformation errors. Although different sources of errors have been reported by clinical studies, no consensus or research has been reported that classifies the source of errors and estimates the PTV margins in the lung and abdominal regions for a relatively large cohort of patients.^9^, ^11^, ^12^, ^18^, ^21^ In this study, the source of errors and uncertainties in the Cyberknife® robotic surgery system are summarized, evaluated, and quantified to determine appropriate margins. In this relation, a large cohort of patients with 159 treatment fractions for 45 patients with lung and abdominal cancer was studied to estimate PTV margins based on two validated methods: Van Herk formula ^22^, ^28^ and uncertainty estimation method.^12^ Additionally, to investigate the effects of errors on the estimated PTV margins, a statistical analysis was also performed.

## 2 MATERIALS AND METHODS

### 2.1 Data source and properties

Tumor motion data, including 45 lung and abdominal cancer patients treated with the Cyberknife synchrony system, were used in this study.^23^ Table 1 presents the features of the case studies, including the tumor sites, the number of case studies, and the treatment fractions.

**TABLE 1:** Features of the case studies, including tumor sites, number of patients, and number of treatment fractions

Area	Tumor sites	Number of patients	Number of treatments
Lung area	Lung apex left	1	3
	Lung hilum	1	4
	Lung hilum left	1	3
	Lung hilum right	2	8
	Lung LLL	2	10
	Lung LUL	11	33
	Lung RLL	5	15
	Lung RML	4	13
	Lung RUL	4	15
Abdomen area	Liver	2	6
	Pancreas	9	28
	Retroperitoneum	3	10
Chest wall	Chest wall	2	6
Internal mammary nodes	Internal mammary nodes	1	5

Abbreviations: LLL, left lower lung; LUL, left upper lobe; RLL, right lower lung; RML, right middle lobe; RUL, right upper lung.

It must be noted that during treatment with the Cyberknife system, the clinical log data are recorded into two concentric coordinate systems (patient and robotic coordinates). Whereas the patient coordinate is based on the same orientation as the imaging coordinate with the origin at the isocrystal position, the robot coordinate or world frame is the robot's location on the pedestal. In addition, the *X*, *Y*, and *Z* in the isocrystal position are in the superior–inferior (S–I) direction, left–right (L–R) direction, and anterior–posterior (A–P) direction, respectively, in Eq. (1).

$$x_{\mathrm{pat}}=0.7081*x_{\mathrm{rob}}+0.7061*y_{\mathrm{rob}}$$

(1) $$y_{\mathrm{pat}}=-0.7081\;*\;x_{\mathrm{rob}}+0.7061\;*\;y_{\mathrm{rob}}$$

$$z_{\mathrm{pat}}=z_{\mathrm{rob},}$$

where the pat and rob are patient and robotic coordinate systems, respectively. Furthermore, the five log files generated via the Cyberknife synchrony system during the treatment are as follows:

1. Markers.log file: contains the raw LED position data received from FlashPoint (Synchrony camera controller).
2. ModelPoints.log file: contains the data used to build the correlation model, including the fiducial marker position (as determined by the imaging system), the position of the external respiratory surrogates (LED markers), and an error in the correlation model.
3. Modeler.log file: contains the correlation model output (position of a target based on the synchrony module), which is updated throughout the treatment from alignment to completion regardless of the beam status at a given time.
4. Predictor.log file: contains the output and error of the prediction of 200 ms, which is necessary to compensate for the inertia between the prediction model and the corresponding correlation model.
5. ERsiData.log file: contains the robot's position in the Robot World coordinate frame and offsets commanded.

### 2.2 Source of Cyberknife system errors

In particular, the results from the previous studies reveal that during treatment with the Cyberknife respiratory tracking system five errors occur: (1) the segmentation error results from the difference between the position of the target (the center of mass, CoM) in digitally reconstructed radiographs (DRRs) and the X‐ray real‐time imaging system; (2) the deformation error results from the difference between identifying the target (CoM) in the X‐ray real‐time imaging system and the planning CT image; (3) the correlation error results from the difference between the output of the correlation model and tumor location as determined by the X‐ray imaging system; (4) the prediction error results from the difference between the prediction algorithm and the corresponding correlation model; and (5) the targeting error results from the difference between the position of the actual target and where the robotic arm in the Cyberknife steered the external beam.^7^, ^9^, ^11^, ^12^, ^13^, ^14^, ^15^, ^16^, ^17^, ^18^, ^19^, ^21^

The ability to distinguish lung tumor density of soft‐tissue in a live X‐ray or DRR image is a challenging issue. As a consequence, it is difficult to calculate the segmentation error throughout treatment.^9^ Jung et al. in their study on the patient‐specific lung phantoms, concluded that total tracking errors are 0.38 ± 0.54 mm in the S–I direction, 0.13 ± 0.18 mm in the L–R direction, and 0.14 ± 0.37 mm in the A–P direction.^15^ It should be noted that in this study, the correlation and prediction errors were minimized by linear fitting of the phantom motion. Since the total tracking error was estimated by determining the 3D distance between the predicted tumor position and the actual tumor position in X‐ray images, the total tracking error could be partly the segmentation error. The maximum displacement of the proposed phantom is in the S–I direction; therefore, in the present work, a segmentation error of 0.38 ± 0.54 mm is assumed for all patients in three directions.

**FIGURE 1:**
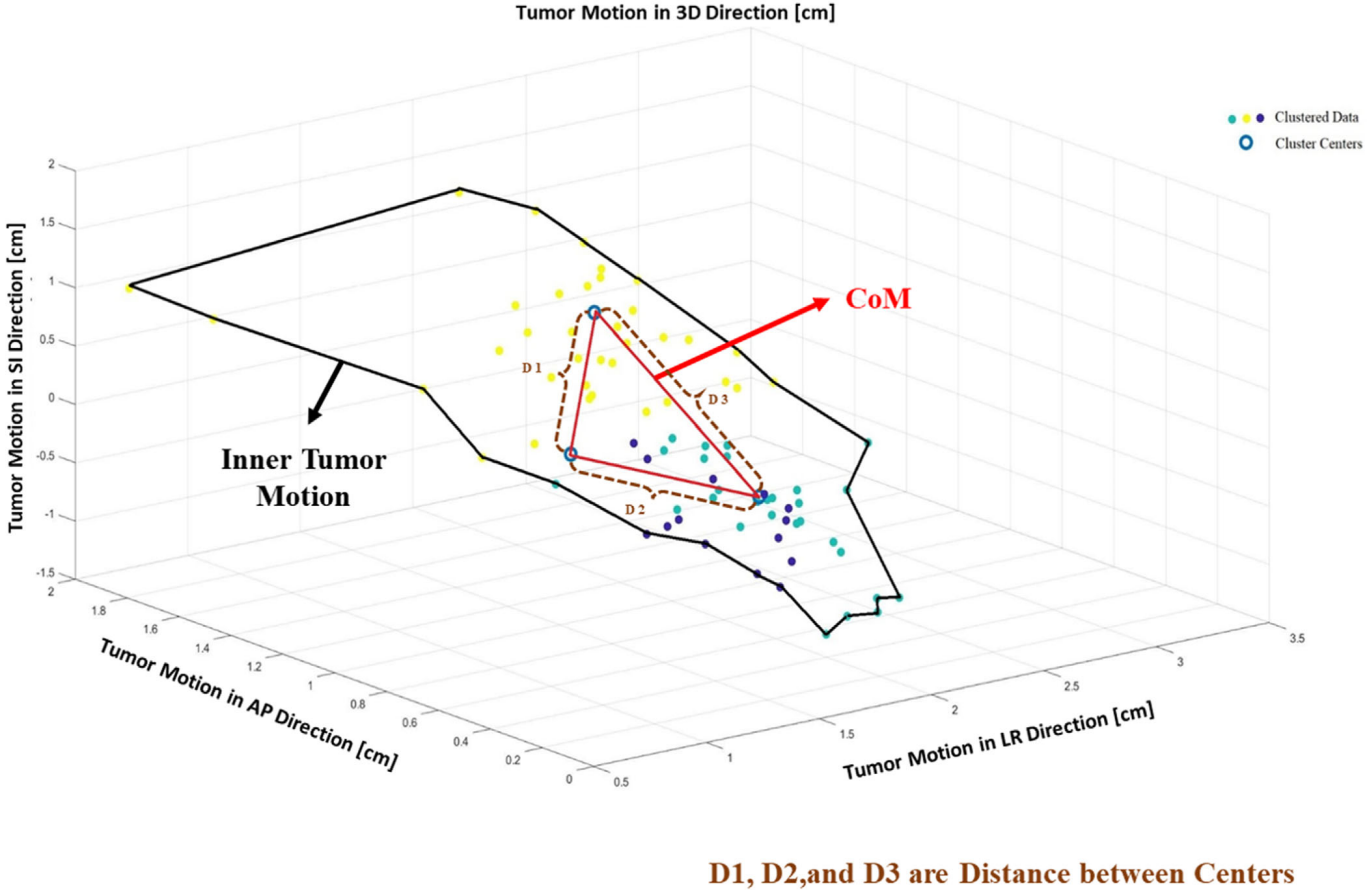
The inner tumor motion obtained from the Model Point log file is represented by the black line. The cluster centers, which are denoted by the blue annulus in the A–P, L–R, and S–I directions, are estimated by the *k*‐means method. The distance between the interconnected annulus is assumed to be the CoM region represented by the red line

The individual patient features, such as tumor motion range and inner tumor motion, have a strong effect on evaluating deformation error. A study conducted by Lu et al. in 2008, which examined 12 lungs and 5 upper abdomen lesions treated with the Cyberknife, showed that 3‐mm and 5‐mm margins are required to compensate for deformation and uncertainty errors, respectively. This study also represents the tumor size and the motion‐range impact on the deformation and uncertainty errors.^24^, ^25^ In this study, by using the same concept, an alternative approach is presented to estimate the deformation error during the treatment. To accomplish this purpose, first, the *k*‐means method is proposed to measure the CoM value in all cases. Subsequently, the deformation error was assessed by analyzing the CoM value in all three directions. The workflow of the proposed approach, which is shown in Figure 1, has the following explanations:

1. The ModelPoint log file also provides information about the fiducial marker position throughout the treatment. Thus, in this perspective, the range of the inner tumor motion as the motion range of the target can be estimated from the fiducial marker movement (represented by the black line in Figure 1). It must be noted that, in the Synchrony dataset, the 3D positions of the centroid of a set of 2–3 fiducials implanted in the tumor were measured using stereoscopic radiography, which is referred to as the tumor position. Each dataset included 40–112 (mean = 62) concurrent tumor and marker localizations at a mean interval of 66 s.
2. To determine the center of the movement in each direction, the *k*‐means clustering method is proposed. This method divides an assigned anonymous dataset into a fixed number of clusters.^26^ Initially, the *k* number (*k* = 3 in this study), which is also called the centroids of the data point, is randomly selected. The results of the training *k*‐means method through the selected centroids are specified as the nearest data points. Based on this perspective, each data point can be assigned to the closest centroids. The positions of the centroids are also recalculated after the data points are assigned. The process of centroid adjustment and classification is repeated until the centroids’ positions are fixed.^27^
3. The *k*‐means method's results, which determine the centroid points in the inner tumor motion, are used to determine the cluster centers in the A–P, L–R, and S–I directions, respectively (marked as the blue annulus in Figure 1).
4. The mean ± standard deviations of the distance among the interconnected annulus points, represented by the red line, are used to calculate the CoM using the equation as follows:
(2) $$\mathrm{CoM}=R=\sqrt{LR^{2}+AP^{2}+SI^{2}.}$$
5. Based on the CoM value, the deformation error can be divided into two groups according to the Smith et al. and Lu et al. studies:^24^, ^25^ (a) if the motion variation in the CoM is less than 2 cm, the uncertainty of the deformation error in all three directions is considered 1.5 mm; (b) if the motion variation in the CoM is greater than 2 cm, the uncertainty of the deformation error in all three directions is considered 2.5 mm.

The accuracy of the correlation model is affected by its properties and also the amount of input/output data, which are analyzed. The input data are acquired by using infrared sensors, and the output data are obtained by using two orthogonal diagnostic X‐ray imaging systems. In this series, the optical camera system monitors the position of three red LED‐based markers attached to a patient's chest, while the two orthogonal diagnostic X‐ray systems monitor markers implanted near the tumor by reading every 30 s or two beams.^7^ The synchrony system uses these data in a correlation model to estimate a tumor's position during the treatment.^23^ The accuracy of the correlation model used in the Cyberknife system has been recorded in the ModelPoints.log file; therefore, analyzing the ModelPoints.log file could provide a proper understanding of the synchrony correlation model.

System latency caused by data acquisition and the robotic system response affects the accuracy of radiation dose delivery. However, the prediction model can estimate the difference between the predictive algorithm and the corresponding modeler points to compensate for the latency. In the Cyberknife system, the real‐time tracking technology is used to overcome 200 to 115 ms latency during radiotherapy. In this relation, the analyzing Predictor.log file provides information about the prediction error related to the predictor model.

The concept of robotic targeting accuracy in the Cyberknife system is related to the accuracy of the 6‐DOF robotic arm to deliver the external beam from a range of unique angles in the 3D workspace to the tumor volume. So, the image registration technique at the beginning of treatment is used to determine the target position in live X‐ray and DRRs images. Based on the target location and the secondary collimator type, different treatment paths are defined between the source positions and the target location in the source‐to‐axis distance of 65–120 cm. Moreover, an optimized path traversal algorithm controls the Cyberknife robot arm movement during the treatment. In practice, however, few treatment paths are selected by the optimized path traversal algorithm.^7^ In this context, the ERsiData.log file in the Cyberknife system provides information about the candidate beams per node and robotic manipulator repeatability during the treatment.

### 2.3 Quantified errors in the Cyberknife system

The difference between the exact and the measured values is known as error. From this perspective, errors can be classified as random or systematic.^28^ Random error or statistical fluctuation originates from the measurement instrument's limitations. Additionally, random error is usually varied from one observation to another and originates from the experimenter's inability to take the same measurement repeatedly.^22^, ^28^ Systematic error, on the other hand, refers to reproducible inaccuracies that are caused by imperfect calibrations of measurement instruments.^28^ In addition, two types of systematic error have a linear response in measurement instruments: (1) offset error (zero setting error) and (2) multiplier error (scale factor error). Since the Cyberknife system is a fully robotic radiotherapy device, evaluating the impact of the errors on the accuracy of the dose delivery is critical. For the first time, the definition of errors was presented by Van Herk et al., in order to quantify the source of errors in radiotherapy. The derived results represent two types of geometrical deviations: treatment execution and treatment preparation variations.^20^ Whereas the treatment execution is day‐to‐day variation (random error), the treatment preparation indicates variation during a single radiotherapy course (systematic error).^20^, ^24^ In this study, based on Van Herk et al., the errors in the Cyberknife tracking system are categorized into random and systematic errors.^22^, ^28^ Based on the evidence currently available, the segmentation, deformation, and targeting errors are classified into systematic errors, while the tracking errors (correlation and prediction errors) include both systematic and random errors.^22^ In the following section, the approach to analyze the clinical log data and the method to evaluate the impact of the errors on the PTV margins are described briefly.

### 2.4 PTV margins based on Van Herk formulation

Based on Van Herk et al., treatment execution (random error) will cause a blurring in the dose distribution, while treatment preparation (systematic error) will cause an unexpected variation in the cumulative dose distribution corresponding to the CTV.^22^ To investigate the effect of the errors on the 3D uniform dose distribution, Van Herk et al. also proposed a method to estimate the PTV margins from the error sources, which can be estimated as follows:

(3) Margin=2.5∑+0.7σ,

where ∑ is the standard deviation (SD) of the treatment preparation and σ represents the SD of the treatment execution. The SD of the systematic error (∑) and the SD of the random error (σ) can be estimated as follows:

(4) $$\sum =\sqrt{\frac{1}{N_{i}-1}\sum_{i=1}^{N_{i}}{\left({\left\langle X\right\rangle }_{i}\right)}^{2}}$$

(5) $$\sigma =\sqrt{\frac{1}{N_{i}}\sum_{i=1}^{N_{i}}{\left(\sigma_{i}\right)}^{2}}$$

(6) $$\sigma_{i}=\sqrt{\frac{1}{N_{ji}-1}\sum_{j=1}^{N_{ji}}{\left(X_{ji}-{\left\langle X\right\rangle }_{i}\right)}^{2}}$$

where ${\left\langle X\right\rangle }_{i}$ is the mean shift for fraction *i* (the mean of correlation–prediction error for fraction *i*), $N_{i}$ the total number of treatment fractions, $\sigma_{i}$ the SD of the correlation–prediction error for fraction *i*, $N_{ji}$ the number of data points in fraction *i*, and $X_{ji}$ is the *j* shift observed for fraction *i*.

The PTV margins recipe in equation (3) must be extended based on five sources of error, leading to equation (7). The margin formulation assumes an isodose coverage of 95% and a probability level of 90%.^26^

(7) $$\begin{array}{ccc} & & \mathrm{Margin}\\ & & \;=\;2.5\sum +\beta \sqrt{\sigma^{2}+\sigma_{\rho }^{2}}-\beta *\sigma \rho \\ & & \;=\;2.5\sqrt{{\left(\sum S\right)}^{2}+{\left(\sum D\right)}^{2}+{\left(\sum C\right)}^{2}+{\left(\sum P\right)}^{2}+{\left(\sum T\right)}^{2}}\\ & & \;+\;\beta \sqrt{\sigma_{C}^{2}+\sigma_{P}^{2}+\sigma_{\rho }^{2}}-\beta *\sigma \rho , \end{array}$$

where ∑*S* is the SD of the segmentation error, ∑*D* is the SD of deformation error, ∑*C* is the SD of correlation error, ∑*P* is the SD of prediction error, ∑*T* is the SD of the targeting error from robot accuracy, $\sigma_{\rho }$ represents the width of the penumbra width of the radiation beam, and β depends on the dose level selected for the dose prescription. In lung SBRT, the penumbra width of the radiation beam is broader than in water and due to the steep dose fall‐off, a dose level of 80% might be applied to the prescription. Under these conditions, σ ρ and β are assumed to be 6.4 mm and 0.84, respectively, for all calculations. For a detailed description, see studies by Van Herk et al.^22^, ^28^

### 2.5 PTV margins based on uncertainty estimation method

In order to estimate the PTV margin, all the main sources of uncertainty must be determined based on the uncertainty estimation method. Therefore, at the first step, all sources of error were evaluated independently to designate them as uncertainty errors. To evaluate the treatment margins, all the main sources of uncertainty were analyzed. Table 2 shows the steps for estimating the entire treatment process with 95% coverage of CTV points at a 95% confidence level. To estimate the expanded uncertainty, the *k* was considered to be two since it assumed a normal PDF. For a detailed description, see studies by Yang Z‐Y et al. and Floriano et al.^9^, ^12^

**TABLE 2:** The calculation steps in the uncertainty estimation method

	**Segmentation**	**Deformation**	**Correlation**	**Prediction**	**Targeting**	**Step process**
Uncertainty	*U_S_* = |μ_s_|+2σ_s_	*U_D_* = |μ_D_|+2σ_D_	*U_C_* = |μ_C_|+2σ_C_	*U_P_* = |μ_P_|+2σ_P_	*U_T_* = |μ_T_|+2σ_T_	Step one
Standard uncertainty^a^	*S_U_S_* = *U_S_*/2	*S_U_D_* = *U_D_*/2	*S_U_C_* = *U_C_*/2	*S_U_P_* = *U_P_*/2	*S_U_T_* = *U_T_*/2	Step two
Combined standard uncertainty	SU _combined_ = $\sqrt{S\_U_{s}^{2}+S\_U_{D}^{2}+S\_U_{C}^{2}+S\_U_{P}^{2}+S\_U_{T}^{2}}$					Step three
Expanded uncertainty^a^	*U*_combined_ = 2* SU _combined_ =$\sqrt{U_{s}^{2}+U_{D}^{2}+U_{C}^{2}+U_{P}^{2}+U_{T}^{2}}$					Step four

*Note*: |μ| as the absolute mean value and σ as the standard deviation (SD).

^a^ Assuming normal PDF.

### 2.6 Statistical analysis

In this study, a systematic design test (SDT) was performed to investigate the effects of input variables on output ones. In other words, the effects of each input, including the effects of segmentation, deformation, correlation, prediction, and robot, and also the effects of couple inputs, including the effects of segmentation and deformation, and the effects of correlation and prediction, were examined. The experiment of the proposed SDT was also performed in three steps. In the first step, each error is considered to be zero; then, the PTV margins are recalculated. This process was also repeated to investigate the effects of couple errors. In the second step, in order to determine the hypothesis of a significant statistical relationship between the recalculated PTV margins (Van Herk formula and the uncertainty estimation method) and tumor displacement, the statistical *F*‐test was performed in all tumor regions and directions. In this test, if the *F* value is greater than a critical value *F_c_* (*F*/*F_c_* > 1) and the *P*‐value < 0.05, a significant relationship between the errors and the PTV margins exists. Finally, the interaction level (original PTV margins–recalculated PTV margins) was calculated and classified to represent the impact levels of each hypothesis assumption. Additionally, another statistical *F*‐test analysis was performed in all tumor locations and directions to check a significant statistical relation between the estimated margins based on the Van Herk formula and the uncertainty estimation method.

## 3 RESULTS

In this work, 45 patients (159 Treatment fractions) were studied. Since it is difficult to distinguish the segmentation error in the live X‐ray or DRR image, in accordance with Jung et al.’s study,^15^ the SD of the segmentation error (∑*S*) is considered to be 0.54 mm in all three directions. To estimate the segmentation error, an alternative approach based on the *k*‐means method is also proposed. The results of the proposed method are presented in Table 3.

**TABLE 3:** Results of the *k*‐means method to estimate the deformation error

		**Mean ± Standard (cm)**					
**Tumor sites**	**Peak‐to‐peak time (s) Mean ± SD**	**S**–**I**	**L**–**R**	**A**–**P**	**CoM**	**Uncertainty of deformation (mm)**	**Standard associated deformation error (mm)** **^a^**
Lung apex left	3.81 ± 0.67	0.09 ± 0.02	0.0 ± 0.01	0.03 ± 0.01	0.094	1.5	0.75
Lung hilum	4.49 ± 0.89	0.72 ± 0.10	0.09 ± 0.08	0.39 ± 0.09	0.823	1.5	0.75
Lung hilum left	3.41 ± 0.72	2.23 ± 0.21	0.02 ± 0.05	0.42 ± 0.11	2.269	2.5	1.25
Lung hilum right	3.71 ± 0.67	3.95 ± 0.33	0.04 ± 0.15	0.74 ± 0.24	4.018	2.5	1.25
Lung LLL	3.14 ± 0.60	4.02 ± 0.30	0.0 ± 0.10	0.87 ± 0.18	4.11	2.5	1.25
Lung LUL	3.73 ± 0.69	2.90 ± 0.21	0.0 ± 0.06	0.36 ± 0.12	2.92	2.5	1.25
Lung RLL	3.56 ± 0.74	4.62 ± 0.73	0.0 ± 0.04	0.38 ± 0.16	4.63	2.5	1.25
Lung RML	4.04 ± 0.96	4.62 ± 0.38	0.07 ± 0.18	0.73 ± 0.26	4.677	2.5	1.25
Lung RUL	4.19 ± 0.80	4.09 ± 0.46	0.0 ± 0.06	0.54 ± 0.17	4.125	2.5	1.25
Liver	3.96 ± 0.71	0.52 ± 0.09	0.0 ± 0.01	0.15 ± 0.04	0.541	1.5	0.75
Pancreas	3.12 ± 0.61	0.60 ± 0.13	0.0 ± 0.04	0.13 ± 0.06	0.613	1.5	0.75
Retro‐peritoneum	3.74 ± 0.80	0.65 ± 0.07	0.01 ± 0.02	0.15 ± 0.05	0.667	1.5	0.75
Chest wall	3.81 ± 0.67	0.09 ± 0.02	0.0 ± 0.01	0.03 ± 0.01	0.094	2.5	1.25
Internal mammary nodes	5.25 ± 1.12	1.69 ± 0.31	0.0 ± 0.12	0.87 ± 0.21	1.9	1.5	0.75

^a^ Assuming normal PDF.

Abbreviations: S–I, superior–inferior; L–R, left–right; A–P, anterior–posterior.

The clinical log data analysis of the ModelPoints.log file, Predictor log file, and ERsiData.log file through the mean value and SD analysis is shown in Table 4. Note that the prediction model in the Cyberknife G3 system uses an adaptive predictor (historic pattern‐matching filter) to compensate for the system latency of 200 ms. Therefore, a larger prediction error is expected; however, this value has been significantly reduced in recent versions of the Cyberknife systems that use the hybrid method.^9^, ^12^, ^21^ In Table 5, for each direction and tumor position, the uncertainty of five errors based on the uncertainty estimation method was reported independently. Note that for tumors with larger displacements, such as lung LLL, lung RLL, liver, and pancreas, the larger uncertainties of correlation and prediction errors were observed. The estimated predictor uncertainty is larger than previously reported results,^9^, ^12^ which may be related to the use of adaptive predictors for compensating the 200 ms system latency in the Cyberknife G3 system. Note that the E2E value is an end result of a plan delivered from all directions. Each beam node may have larger errors on each position (the reported results in Table 4). But at the end, not each small error is important because some errors cancel each other out over the course of the treatment. Therefore, the targeting error is considered form the E2E tests.^29^, ^30^ For a well‐calibrated Cyberknife system, the results of the E2E tests typically represent a static level of 0.3–0.7 mm.^29^ In this study, the estimated result of the E2E tests of Wong et al.^29^, ^30^ was used in all three directions (0.5 ± 0.3 mm).

**TABLE 4:** The mean value and standard deviation of correlation, prediction, and robotic errors

	**Mean value ± standard deviation of correlation error (mm)**			**Mean value ± standard deviation of prediction error (mm)**			**Mean value ± standard deviation of robotic error (mm)**		
**Tumor sites**	**S**–**I**	**L**–**R**	**A**–**P**	**S**–**I**	**L**–**R**	**A**–**P**	**S**–**I**	**L**–**R**	**A**–**P**
Lung apex left	−0.02 ± 0.98	−0.08 ± 0.34	0.12 ± 1.00	0.18 ± 0.53	0.14 ± 0.35	0.14 ± 0.40	−1.90 ± 0.62	−0.82 ± 0.66	−1.13 ± 0.62
Lung hilum	−0.20 ± 1.38	−0.17 ± 2.05	−0.32 ± 1.49	0.32 ± 0.60	0.58 ± 0.88	0.58 ± 0.78	0.25 ± 1.25	−0.13 ± 2.31	0.11 ± 2.54
Lung hilum left	−0.10 ± 0.58	0.09 ± 0.60	0.10 ± 0.84	0.26 ± 0.21	0.27 ± 0.27	0.24 ± 0.24	0.25 ± 1.25	−0.13 ± 2.31	0.11 ± 2.54
Lung hilum right	−0.09 ± 0.44	0.16 ± 0.71	0.05 ± 0.61	0.16 ± 0.15	0.43 ± 0.45	0.42 ± 0.46	−0.27 ± 0.78	−2.04 ± 1.56	−1.06 ± 1.61
Lung LLL	−0.09 ± 1.81	0.03 ± 1.39	−0.07 ± 1.42	0.56 ± 0.76	0.60 ± 0.86	0.59 ± 0.93	−1.80 ± 2.33	0.92 ± 2.20	2.24 ± 2.12
Lung LUL	−0.09 ± 1.12	0.00 ± 0.90	0.00 ± 0.90	0.40 ± 0.56	0.35 ± 0.45	0.41 ± 0.51	−0.14 ± 1.83	−0.13 ± 1.56	−0.51 ± 1.85
Lung RLL	−0.12 ± 0.69	0.17 ± 1.23	−0.03 ± 0.71	0.29 ± 0.43	0.83 ± 1.14	0.73 ± 0.79	−1.74 ± 1.27	−1.96 ± 2.80	−0.97 ± 2.55
Lung RML	0.01 ± 0.65	0.02 ± 0.64	0.03 ± 0.62	0.29 ± 0.43	0.41 ± 0.41	0.33 ± 0.36	−0.63 ± 0.93	−1.28 ± 1.48	0.95 ± 1.01
Lung RUL	−0.01 ± 1.05	0.09 ± 1.23	0.14 ± 1.16	0.37 ± 0.51	0.49 ± 0.63	0.41 ± 0.62	−0.03 ± 1.25	−2.49 ± 2.08	1.30 ± 1.87
Liver	0.06 ± 0.49	0.13 ± 1.02	−0.40 ± 1.08	0.56 ± 0.65	0.71 ± 0.71	0.50 ± 0.54	−1.00 ± 2.10	0.39 ± 2.71	−1.98 ± 2.26
Pancreas	0.07 ± 0.96	0.00 ± 0.93	0.01 ± 0.96	0.32 ± 0.43	0.51 ± 0.56	0.49 ± 0.52	−0.18 ± 1.14	−0.54 ± 1.94	−0.06 ± 1.88
Retro‐peritoneum	0.00 ± 0.25	0.03 ± 0.40	−0.02 ± 0.24	0.08 ± 0.11	0.11 ± 0.13	0.13 ± 0.16	−0.12 ± 0.34	0.71 ± 0.57	0.30 ± 0.59
Chest wall	−0.01 ± 0.59	−0.15 ± 0.50	0.07 ± 0.49	0.23 ± 0.33	0.16 ± 0.42	0.16 ± 0.52	−0.66 ± 1.18	−0.89 ± 1.21	0.96 ± 1.18
Internal mammary nodes	−0.10 ± 0.83	0.07 ± 0.44	−0.16 ± 0.77	0.11 ± 0.15	0.11 ± 0.16	0.08 ± 0.11	−2.90 ± 0.94	3.03 ± 0.94	−0.49 ± 0.58

Abbreviations: S–I, superior–inferior; L–R, left–right; A–P, anterior–posterior.

**TABLE 5:** The uncertainty of segmentation error, deformation error, correlation error, prediction error, and targeting error for different tumor locations in each direction

			Correlation Uncertainty (mm)			Prediction Uncertainty (mm)			
Tumor sites	Segmentation Uncetaity (mm) ^a^	Deformation Uncertainty (mm) ^a^	S–I	L–R	A–P	S–I	L–R	A–P	Targeting Uncertainty (mm) ^a^
Lung apex left	1.46	2.5	1.99	0.77	2.12	1.24	0.84	0.94	0.78
Lung hilum	1.46	2.5	2.98	4.49	3.35	1.51	2.35	2.15	0.78
Lung hilum left	1.46	2.5	1.33	1.30	1.79	0.68	0.80	0.72	0.78
Lung hilum right	1.46	2.5	0.98	1.61	1.31	0.46	1.32	1.35	0.78
Lung LLL	1.46	2.5	3.86	3.04	3.09	2.09	2.32	2.45	0.78
Lung LUL	1.46	2.5	2.43	1.97	1.96	1.52	1.26	1.43	0.78
Lung RLL	1.46	1.5	1.53	2.69	1.55	1.14	3.12	2.31	0.78
Lung RML	1.46	1.5	1.38	1.39	1.33	1.14	1.24	1.04	0.78
Lung RUL	1.46	1.5	2.29	2.64	2.53	1.40	1.75	1.64	0.78
Liver	1.46	1.5	1.05	2.28	2.56	1.87	2.12	1.58	0.78
Pancreas	1.46	2.5	2.06	2.07	2.05	1.30	1.63	1.52	0.78
Retro‐peritoneum	1.46	1.5	0.57	0.86	0.72	0.30	0.37	0.49	0.78
Chest wall	1.46	1.5	1.24	1.15	1.10	0.90	1.01	1.20	0.78
Internal mammary nodes	1.46	1.5	1.77	2.28	1.70	0.42	0.42	0.30	0.78

^a^ In all three directions.

Abbreviations: S–I, superior–inferior; L–R, left–right; A–P, anterior–posterior.

This study used the two validated approaches to calculate the required PTV margins; Van Herk formulation and uncertainty estimation method. According to Table 6, the required PTV margin in the lung area based on the Van Herk formulation was 4.39 ± 0.96 (mm), 4.68 ± 1.13 (mm), and 4.47 ± 0.93 (mm) in the S–I, L–R, and A–P directions, respectively. This value based on the uncertainty estimation method was 4.53 ± 1.11 (mm), 4.89 ± 1.33 (mm), and 4.69 ± 1.19 (mm) in the S–I, L–R, and A–P directions, respectively. Also, performing the statistics *F*‐test analysis demonstrates that there is no difference between the two presented approaches.

**TABLE 6:** The mean ± SD of the calculated PTV margins based on Van Herk formula and the uncertainty estimation method in each direction for different tumor locations

	PTV margin based on Van Herk formula (mm)			PTV margin based on uncertainty estimation method (mm)		
Tumor sites	S–I	L–R	A–P	S–I	L–R	A–P
Lung apex left	4.14 ± 1.01	3.56 ± 0.33	4.05 ± 0.90	4.01 ± 0.98	3.28 ± 0.26	3.93 ± 0.75
Lung hilum	4.40 ± 0.87	5.12 ± 1.01	4.74 ± 1.08	4.61 ± 0.77	5.93 ± 0.88	5.22 ± 0.82
Lung hilum left	3.62 ± 0.31	3.62 ± 0.07	3.76 ± 0.08	3.37 ± 0.20	3.38 ± 0.02	3.57 ± 0.08
Lung hilum right	3.55 ± 0.33	3.97 ± 0.85	3.81 ± 0.69	3.25 ± 0.25	3.81 ± 0.71	3.63 ± 0.50
Lung LLL	4.87 ± 1.70	4.61 ± 1.13	4.75 ± 1.55	5.13 ± 1.35	5.02 ± 1.03	5.06 ± 1.62
Lung LUL	4.30 ± 1.19	4.13 ± 1.21	4.13 ± 1.26	4.29 ± 1.12	3.97 ± 1.01	4.03 ± 0.90
Lung RLL	2.95 ± 0.70	3.67 ± 1.96	3.01 ± 0.91	3.08 ± 0.55	4.81 ± 1.85	3.68 ± 0.76
Lung RML	2.97 ± 0.65	2.78 ± 0.69	2.70 ± 0.50	2.97 ± 0.53	3.0 ± 0.51	2.87 ± 0.39
Lung RUL	3.40 ± 1.52	3.97 ± 1.99	3.37 ± 0.97	3.71 ± 1.51	4.15 ± 2.22	3.90 ± 1.04
Liver	2.88 ± 0.60	3.26 ± 0.51	3.22 ± 1.11	3.07 ± 0.50	3.92 ± 0.41	3.88 ± 1.15
Pancreas	4.04 ± 0.71	4.10 ± 0.39	4.16 ± 1.05	4.0 ± 0.69	4.08 ± 0.56	4.07 ± 0.89
Retro‐peritoneum	2.34 ± 0.12	2.46 ± 0.30	2.38 ± 0.23	2.35 ± 0.08	2.48 ± 0.23	2.48 ± 0.28
Chest wall	2.65 ± 0.40	2.73 ± 0.60	2.92 ± 0.98	2.81 ± 0.46	2.91 ± 0.62	3.07 ± 1.07
Internal mammary nodes	2.76 ± 0.49	2.42 ± 0.10	2.70 ± 0.55	2.93 ± 0.41	2.47 ± 0.05	2.88 ± 0.44

Abbreviations: S–I, superior–inferior; L–R, left–right; A–P, anterior–posterior.

The error interaction levels, including segmentation, deformation, correlation, prediction, robotics, segmentation and deformation, and correlation and prediction errors, on the estimated PTV margins based on Van Herk formula were examined in the lung region (lung LLL, lung LUL, lung RLL, lung RML, and lung RUL) and the abdominal region (liver, pancreas, and retroperitoneum). In this regard, the *F*‐test results of the SDT represent all tumor regions that pass the conditions mentioned in Section 2.6 (Table S1.xlsx).

**FIGURE 2:**
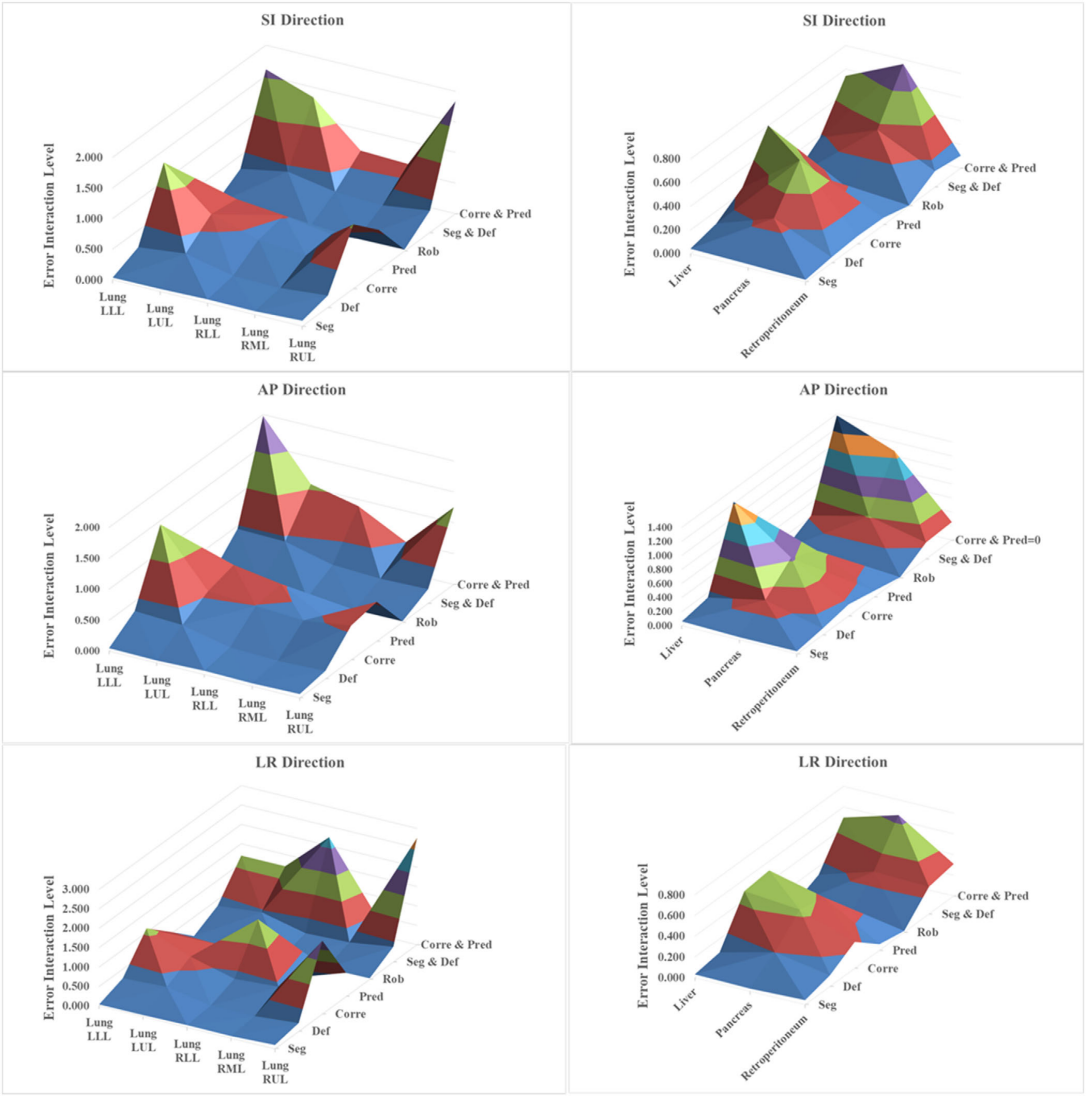
The results of systematic design for each tumor region in lung and abdomen area based on the error interaction level on the calculated PTV margin (Van Herk formulation). Note that the vertical axis is the error interaction level (original PTV margins–recalculated PTV margins)

As shown in Figure 2, depending on the tumor locations and directions, different interactions levels can be suggested, ranging from low to very high. In this figure, different colors are used to represent the interaction levels (low to very high impact). Additionally, the vertical axis is the error interaction level (original PTV margins–recalculated PTV margins). Overall, the targeting error due to robot repeatability was small and can be ignored in the overall margin with synchrony, while the correlation and prediction errors have a significant impact on the estimated PTV margins. Depending on the tumor location, the correlation or prediction error has a moderate to high impact on the PTV margins in all three directions. It must be noted that in the regions with larger shifts, such as lung LLL or liver, the error interaction values are higher than in other regions.

## 4 DISCUSSION

In terms of respiratory motion management, a number of SBRT techniques can be applied to decrease the irradiated volume. Among the available SBRT techniques, the real‐time respiratory tracking system included within the Cyberknife system exhibits a high degree of accuracy in the delivery of the radiation dose. Therefore, in order to investigate the accuracy of radiation beam delivery, the assessment of the respiratory tracking system is essential. In this regard, several studies have sought to classify the source of errors or proposed an approach for calculating the PTV margins through the source of errors in the Cyberknife respiratory tracking system.^11^, ^12^, ^13^, ^14^, ^15^, ^16^, ^17^, ^18^, ^19^, ^20^ For instance, Van Herk et al. presented an approach for calculating the PTV margins based on the source of errors during the treatment.^22^ Using the same method, Descovich et al. calculated the ITV‐to‐PTV margins for 24 lung cancer patients without the respiratory tracking system. In that particular cohort, in order to cover the ITV‐to‐PTV margins during treatment with the Cyberknife system, the 6.81 mm, 4.42 mm, and 4.67 mm margins in the S–I, L–R, and A–P directions, were proposed, respectively.^11^ In addition, Floriano et al. proposed an alternative method for estimating the tumor margins based on the uncertainty level. Their results indicated that 5 mm margins in all three directions represented appropriate PTV margins for 16 lung cancer patients when the Cyberknife treatment system (FTT system) was used.^12^ More recently, Yang et al. quantified the clinical accuracy of the Cyberknife system (XLT system) in calculating the PTV margins based on Van Herk et al.’s formalism and uncertainty data. They determined that 4 mm PTV margins were sufficient to provide more than 95% coverage in all three directions in 22 lung cancer patients.^9^ The present study sought to address the limitations of prior studies by considering a large cohort of patients (159 treatment fractions for 45 lung and abdominal cancer patients). Through analyzing these data, five different sources of errors, including segmentation, deformation, correlation, prediction, and targeting errors, were also identified in the Cyberknife respiratory tracking system.

The degree of tumor deformation, however, is strongly affected by the inner tumor motion, tumor motion variation, and tumor motion speed. Smith et al. found that the deformation error in the lung region increases by increasing the distance from the CoM.^25^ In addition, Lu et al. analyzed the inner tumor variation of 12 lung patients and determined 3 mm margins to be sufficient to cover the CTV.^24^ In the present study, an alternative approach based on the *k*‐means method is proposed to estimate the deformation uncertainty based on the inner tumor motion data, which the results presented in Table 3, clearly indicate that 2.5 mm and 1.5 mm margins in all three directions provide sufficient coverage of the tumor motion in the lung area and abdomen area, respectively.

The results of the analysis concerning the correlation, prediction, and robotic errors in terms of the errors and uncertainties are presented in Tables 4 and 5, respectively. As shown in Table 4, the largest error in the Cyberknife respiratory tracking system is related to the targeting error, which can be decreased by considering a monthly E2E test. In the present study, the result of the E2E test was 0.5 ± 0.3 mm in all three directions, which is in good agreement with the result recorded by Wong et al.^29^, ^30^ The reported data also show that in order to provide proper PTV margins during treatment, the tracking errors (correlation and prediction errors) must be considered. In this relation, Yang et al. recommended that during the treatment with the Cyberknife XLT system, in order to provide 100% coverage of the PTV margins in all three directions, the tracking errors must be less than 4 mm. Moreover, they noted that the highest error was related to the correlation model.^9^ Additionally, according to Table 5, the estimated correlation model uncertainty is in good agreement with the previously reported studies,^9^, ^12^ while the prediction uncertainty was larger because the adaptive predictor was used to compensate for the system latency of 200 ms in the Cyberknife G3 system. Interestingly, Radixact currently employs the synchrony respiratory tracking technology, which was available in the CyberKnife system, to correct for tumor motion induced by breathing. In this context, B Yang et al. performed a study in 2021 to evaluate the modeling accuracy of the Radixact and CyberKnife's synchrony systems from the different respiratory motion traces.^31^ Radixact demonstrated a smaller root‐mean‐square error (RMSE) than CyberKnife, in a case with a smaller amplitude and a linear correlation between breathing and target motions. Overall, increasing the motion amplitude and the cycle time show that the RMSE of both systems increases and decreases, respectively. Additionally, under unfavorable conditions, the Radixact synchrony system's modeling accuracy is better than the CyberKnife synchrony systems.^31^

The results of the analysis of the PTV margins based on the source of errors and uncertainties are presented in Table 6. Based on these data, the PTV margins were estimated using two validated methods: the Van Herk formula and the uncertainty estimation method. In addition, the margin calculated with the Van Herk formulation was slightly larger than the margin estimated with the uncertainty method. This slight discrepancy could be related to the differences between the two methods for determining the margin. Overall, the results obtained by these two methods are consistent with the results of other studies.^9^, ^11^, ^12^ Concerning the *P*‐value, in the liver region, by considering the estimated PTV margins by the two methods (Van Herk formula and the uncertainty estimation method) are (*P* = 0.039 and *P* = 0.036) in the S–I direction, (*P* = 0.028 and *P* = 0.027) in the L–R direction, and (*P* = 0.022 and *P* = 0.021) in the A–P direction. The results of the estimated PTV margins, shown in Table 6, also reveal three important findings. First, in the lung region, a margin of 5 mm is required in all three directions for coverage greater than 95%. Second, in the liver region, in order to provide proper PTV margins above 95%, a margin of 4 mm in the S–I direction, a margin of 4.5 mm in the L–R direction, and a margin of 5 mm in the A–P direction are sufficient. Third, in the pancreas region, a 5 mm margin in the S–I direction, a 4.5 mm margin in the L–R direction, and a 5 mm margin in the A–P direction are required to achieve coverage greater than 95%. A comparison of the present results concerning the lung and abdomen areas with other studies reveals that an overall 4–5 mm and 5 mm margin in the lung area and abdomen area, respectively, could provide an excellent reference point for selecting the PTV margins in all three directions. Since our reported results (Table 6) were the general option, the PTV margins should generally be assessed patient by patient. Also, the residual errors from incorrect correlation and prediction models must be taken into account during the treatment. Some efforts have been taken in this regard to make this selection criterion as objective as feasible.^32^ Overall, uncorrected systematic rotation and deformation errors (highly dependent on tumor shape and location and tumor motion range), systematic and random motion modeling errors (highly dependent on tumor motion range and LED positions), system accuracy (E2E), and latency errors from motion prediction are the main sources of errors that should be monitored during the treatment.^32^ Furthermore, because the correlation and prediction errors are the same in target tracking either fiducial or fiducial‐free mode, there is no significant difference between the two modes (FTT or XLT); however, this slight discrepancy might be due to the impact of segmentation or deformation error.^15^ Overall, it should be noted that in order to provide suitable PTV margins, the tumor location and the direction of maximum displacement must be considered.

## 5 CONCLUSION

Although there have been relatively few research studies that estimated the PTV margins through treatment, the PTV margins were estimated for a reasonably large cohort of 33 lung cancer patients (106 treatment fractions) and 17 abdominal cancer patients (55 treatment fractions). This study essentially demonstrates that adequate coverage of the PTV margins for all three directions in the lung and abdominal areas could be provided by 4–5 mm and 5 mm margins, respectively. One of the limitations of this study is using the segmentation error based on previous works, which will be resolved in future studies.

## CONFLICT OF INTEREST

The authors declare that there is no conflict of interest that could be perceived as prejudicing the impartiality of the research reported.

## AUTHOR CONTRIBUTION

All listed authors contributed to the study and to drafting the article.

## FUNDING INFORMATION

None.

## ETHICS STATEMENT

The Georgetown Institutional Review Board has approved the use of these data for research purposes (IRB‐2005‐309).

## Supporting information



Supporting Information
